# Role of LET and chromatin structure on chromosomal inversion in CHO10B2 cells

**DOI:** 10.1186/2041-9414-5-1

**Published:** 2014-01-28

**Authors:** Ian M Cartwright, Matthew D Genet, Akira Fujimori, Takamitsu A Kato

**Affiliations:** 1 Department of Environmental and Radiological Health Sciences, Colorado State University, 1618 Campus Delivery, Fort Collins 80523, USA; 2 National Institute of Radiological Sciences, Research Center for Charged Particle Therapy, International Open Laboratory, Anagawa Inage, Chiba 263-8555, Japan

**Keywords:** Inversions, DNA damage, DNA repair, Cytogenetics

## Abstract

**Background:**

In this study we evaluated the effect of linear energy transfer (LET) and chromatin structure on the induction of chromosomal inversion. High LET radiation causes more complex DNA damage than low LET radiation; this “dirty” damage is more difficult to repair and may result in an increase in inversion formation. CHO10B2 cells synchronized in either G1 or M phase were exposed 0, 1, or 2 Gy of 5 mm Al and Cu filters at 200 kVp and 20 mA X-rays or 500 MeV/nucleon of initial energy and 200 keV/μ m Fe ion radiation. In order to increase the sensitivity of prior techniques used to study inversions, we modified the more traditional Giemsa plus fluorescence technique so that cells were only allowed to incorporate BrdU for a single cycle verses 2 cycles. The BrdU incorporated DNA strand was labeled using a BrdU antibody and an Alexa Fluor 488 probe. This modified technique allowed us to observe inversions smaller than 0.6 megabases (Mb).

**Results:**

In this study we have shown that high LET radiation induces significantly more inversions in G1 cells than in M phase cells. Additionally, we have shown that the sizes of the induced inversions not only differ between Fe ion and X-rays, but also between G1 and M phase cells exposed to Fe ions.

**Conclusion:**

We have effectively shown that both radiation quality and chromosome structure interact to alter not only the number of inversions induced, but also the size of the inversions.

## Background

Chromosome inversions, along with several other symmetrical rearrangements, are commonly thought to cause a rearrangement of the chromosome without resulting in the loss of genomic information. There are two types of inversions; pericentric inversions, involving the centromere, and paracentric inversions, located on a single arm of the chromatid. Since pericentric inversions involve the centromere they can be detected by simple karyotyping with Giemsa staining if the breaks occur asymmetrically across the centromere [1,2]. Paracentric inversions, however, do not cause a visual structural change in the chromosome, thus causing them to be extremely difficult to evaluate without using classical Giemsa banding or current mBAND techniques. Both of these techniques are limited by the size of detectable inversions. Additionally, mBAND is extremely costly and limited in the range of which it can stain [1,3-6]. It has been shown that high linear transfer energy (LET) radiation, such as charged particle radiation, creates more complex DNA damage than X-ray and gamma radiation. This complex damage lends itself to an increase in chromosomal aberrations, including chromosome inversion [3,7-9]. Chromosome inversions are a potentially important chromosomal aberration because the cell undergoes genetic recombination and loses no genomic material; this damage can be passed on to a daughter cell leading to a potential mutation. Chromosomal inversion may have played a key role in the evolution of the primate genome. There have been a total of 1,576 putative regions of inverted orientation identified, covering more than 154 Mb of DNA [10]. Of these inversions, it is believed that the pericentric inversions have played the largest role in speciation and evolution [11]. Additionally, it has been observed that radiation-associated papillary thyroid cancer can be caused by a rearrangement of the RET gene due to an inversion. It was shown that the common RET/PTC1 rearrangement is an inversion on chromosome 10 where RET and H4 are juxtaposed. These two genes, which are 30 Mb apart and roughly separated by 1–3 μm, were brought together by a single track of X-ray radiation [12]. Finally, it has been shown that inversions can cause genomic instability by causing a fragile site in the DNA that could lead to future DSBs or translocation [13]. This leads us to believe that despite the fact little to no DNA information is lost, chromosomal inversions have the possibility to cause potential mutagenesis of the irradiated cells, whether this is through direct rearrangement of regulatory elements or through the creation of fragile sites.

In 2013 the Bailey et al. utilized a directionally orientated single stranded probe to identify radiation induced inversion on human chromosome 3 and 10, this stain allowed for visualization of inversions as small as 1 Mb [14]. In our study we altered the modified Giemsa plus Fluorescence (FPG) approached utilized by Bedford’s group by only incorporating BrdU for a single cycle and labeling the BrdU incorporated DNA strand with a BrdU antibody and an Alexa Fluor 488 probe [15]. By utilizing this modified staining protocol we were able to observe extremely small inversions, as small as 0.6 Mb, over all 21 chromosomes of the CHO10B2 genome. This increase in sensitivity allows us to better understand the extent of the induced inversion in irradiated cells. In this study we have shown that both the quality of the radiation and the cell cycle are involved in not only the number of inversions formed, but also the size of these inversions.

## Results

### Validation of BrdU staining protocol

**Figure 1:**
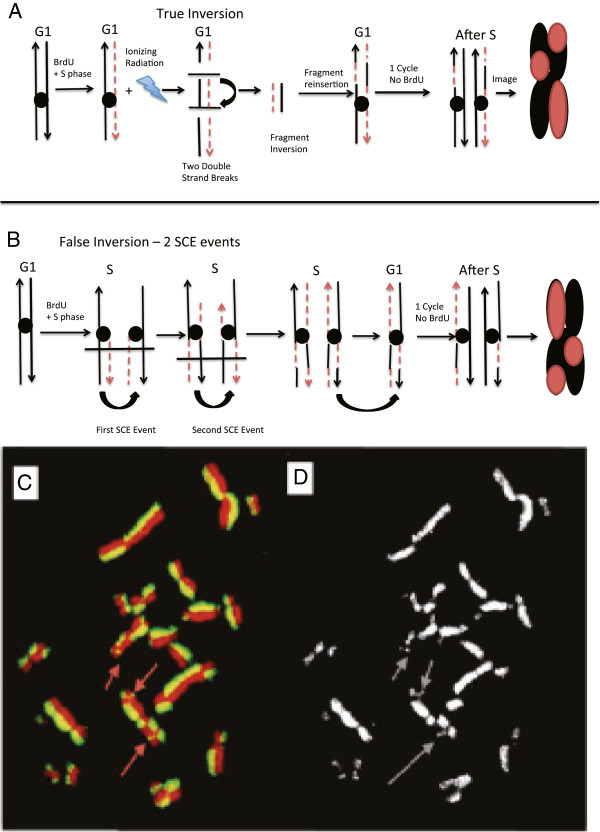
**A method for Inversion detection with BrdU.** Panel **A** depicts how a true inversion is formed. Panel **B** depicts how 2 SCE events can cause a false inversion. Panel **C**. depicts a 21 chromosome CHO10B2 spread exposed to 2 Gy Fe ion radiation and stain with DAPI (pseudo-colored red) and Anti-BrdU (green). The arrows indicate micro inversions, which are considered true inversions; roughly 1–10 Mb. Panel **D**. is the black and white image of the Anti-BrdU image used to measure inversions pixel intensity.

**Figure 2:**
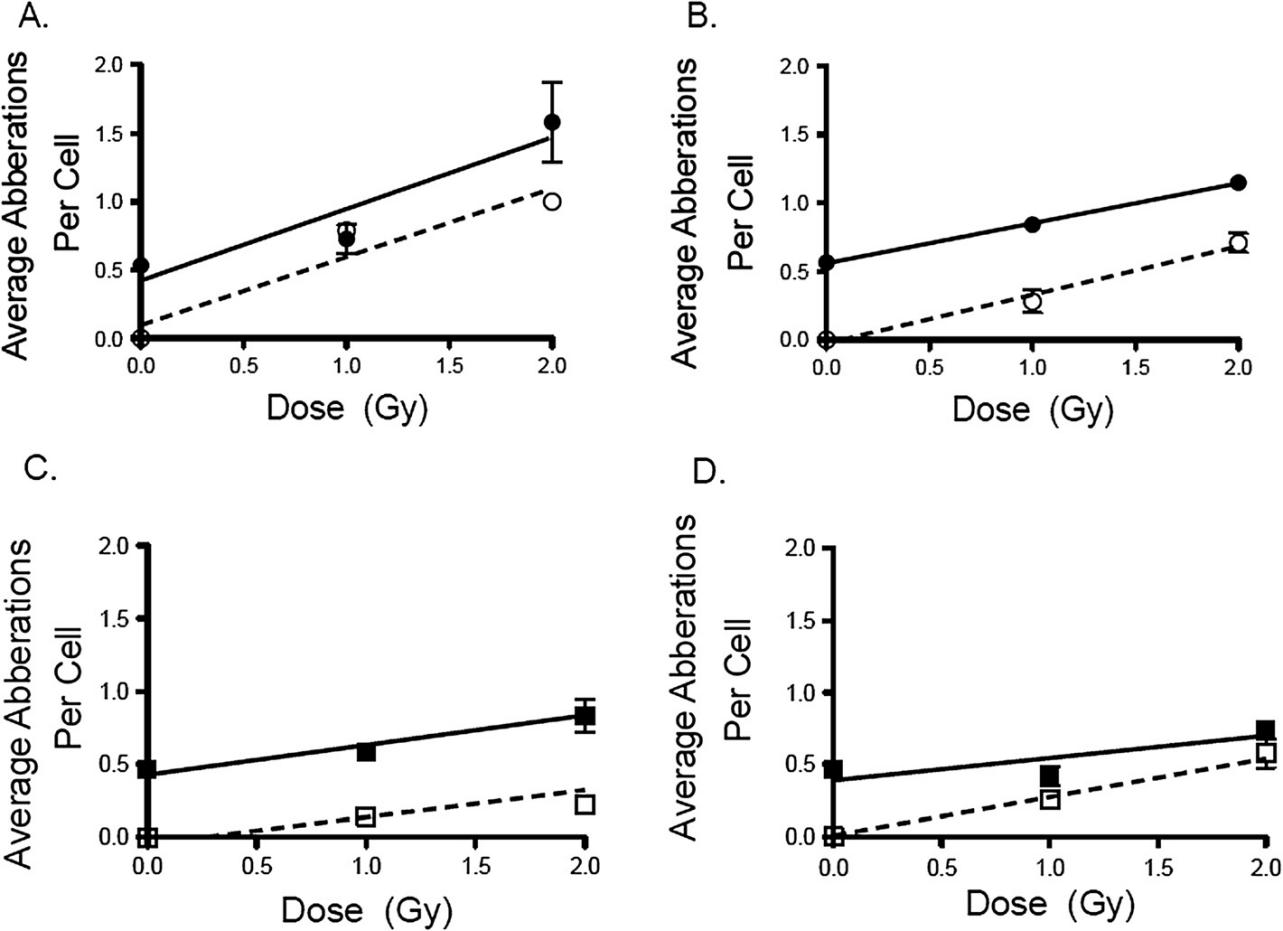
**Dose response curves comparing the induced micro inversions to the observed centric/acentric rings.** 100 spreads at each dose were analyzed. Panels **A** and **B** are G1 and M phase, respectively, exposed to 200 keV/μm Fe ion radiation. Panels **C** and **D** are G1 and M phase, respectively, exposed to 200 kVp X-ray. ● and ○ indicate observed micro inversions and centric/acentric rings respectively for Fe ion exposed cells and ■ and □ indicate observed micro inversions and centric/acentric rings respectively for X-ray exposed cells. The error bars are the standard error of the mean.

Initially, we had to differentiate between true inversions and false inversion. As seen in panel A of Figure 1, true inversions are created during the G1 stage when the cells were irradiated. Panel B of Figure 1 shows how 2 Sister Chromatid Exchange (SCE) events can cause a false inversion; these look exactly the same as a true inversion when imaged. The only way to discriminate between false inversions and true inversions was to calculate the predicted number of false inversions caused by the background SCE events. It was noted that the background level of total inversions, both true and false, was roughly two exchanges per cell. We accounted for the background level of false inversion by calculating the likelihood of a chromosome experiencing 2 SCE events. As seen in Table 1 we used a Poisson distribution, $p\left(x\right)=\frac{\lambda^{x}e^{-\lambda }}{x!}$ were e = 2.71828, *λ* = 0.5381, and x = the variable, to calculate the predicted likelihood of having 1 SCE within a chromosome, at least 2 SCE within a chromosome, or 2 SCE events within 15 Mb of each other on a single chromatid. The R-value is the average number of SCE events per chromosome. Using this we calculated the cumulative frequency, 0.102, and calculated the predicted number of SCE events per 21 chromosomes. To calculate the predicted number of 2 SCE events within 15 Mb, we averaged the chromosome size, 117 Mb, and determined there was a likelihood,15/116 chance, of having a second SCE event within 15 Mb of the first. We multiplied these odds against the odds of 2 SCE occurring on a single chromatid to estimate the predicted value of 2 SCE within 15 Mb. It was noted that the predicted number of false inversions matched extremely well with the observed false inversions at 0 Gy. Based on our calculations we believed that we could effectively identify true inversions by only counting inversions that were roughly 15 Mb in size, which is roughly the width of a chromatid. To ensure that we were observing true inversions using this size exclusion method of counting inversions, we compared our results to previous research which had shown that in normal human fibroblasts (AG1521 cells) the number of induced rings equaled the number of induced inversions at a specific doses [15]. We compared the number of micro inversions to the number of rings at each dose. As seen in Figure 2 the induction rate for all cells exposed to X-ray and Fe ion were statistically similar for rings and micro inversions despite the observed numbers being different. This shows that by excluding inversions larger than the width of a chromatid we were observing true inversion events and excluded most of the false inversions. Additionally, based on prior research, when cells were irradiated in the G1 phase after BrdU was incorporated the level of true SCE did not increase and that all intestinal exchanges events above background were attributed to chromosomal exchange aberrations [15].

**Table 1:** This table outlines the predicted and observed values for a chromosome to contain at 1 SCE within a chromosome, at least 2 SCE within a chromosome, or 2 SCE events within 15 megabases

	**Average’Number’of’ SCE’per’21’ Chromosomes**	**Average’Number’of’ Chromosomes’with’1’ SCE’**	**Number’of’ Chromosomes’with’ at’least’2’SCE’**	**Number’of’ Chromosomes’with’2’ SCE’within’15’Mb**
Predicted’Number’	N/A’	N/A’	2.14’	0.549’
Observed’Number’	11.3’	0.513’	2.2’	0.52’

The predicted values where calculated using a Poisson distribution, using the cumulative odds for each value.

### Effect of radiation quality and cell cycle on Induction of chromosomal inversion

**Figure 3:**
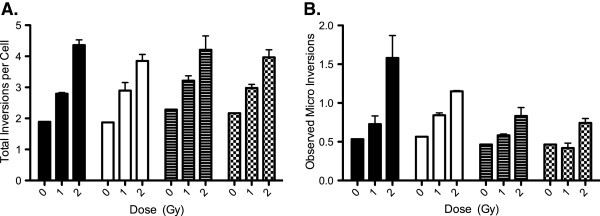
**Total inversions and micro inversions.** This figure depicts the total inversions in panel **A** and the average observed micro inversions in panel **B** observed in both G1 and M phase cells exposed to 0, 1, or 2 Gy of 200 keV/μm Fe ions or 200 kVp X-ray. The solid black is Fe G1 inversions, the white bars are Fe M inversions, the striped bars are X-ray G1 inversions, and the checkered bars are X-ray M inversions. The error bars are the standard error of the mean.

To measure the effect of radiation quality and cell cycle on the induction of inversions we analyzed the induced inversions at 2 Gy of Fe ion or X-ray radiation in both G1 and M phase cells. In Figure 1C and D, we see a 21 chromosome CHO10B2 spread stained using our single cycle BrdU and antibody approach. The arrows indicate inversions, which we classified as micro inversions. As seen in Figure 3A, the total number of induced inversions were statistically similar for both G1 and M phase cells exposed to Fe ions and X-ray. We further counted the number of induced micro inversions and compared the results. As seen in Figure 3B, Fe ions were able to induce more micro inversion in G1 cells than X-rays were able to induce in both M phase and G1 cells, a p-value of 0.0001 and 0.0001 respectively. G1 cells exposed to Fe ions also produced statistically more micro inversions than M phase cells at the same dose, a p-value of 0.0064. Additionally, we observed that the M phase Fe ion exposed cells produced statistically more micro inversion than M phase cells exposed to X-rays, a p-value of 0.0079. Both G1 and M phase cells exposed to X-rays had statistically similar induced inversions, a p-value of 0.381. All results remained the same when the background level of false inversion was subtracted from the observed values.

### Analysis of inverted fragment sizes

**Figure 4:**
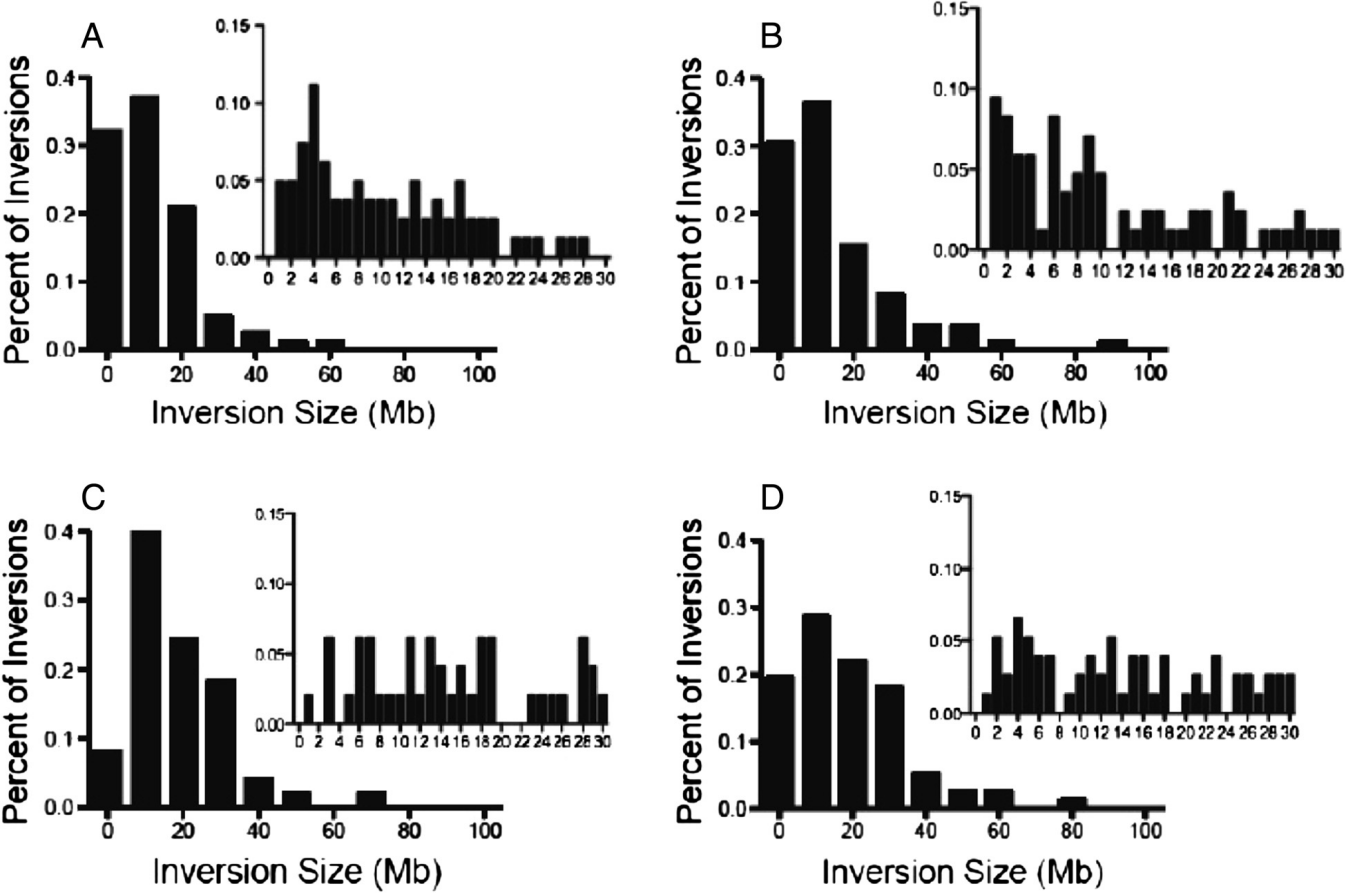
**The size distribution of inversions in cells exposed to 2 Gy of either Fe ion or X-ray radiation.** Panels **A** and **B** are G1 and M phase cells exposed to Fe ion radiations. Panels **C** and **D** are exposed to X-ray. The value of 0 indicts values 0–20 megabases in the main figures. The inlays are a small segment of the original figure highlighting the smaller than 30 megabases. The value of 2 in the inlay indicts values of 0–2 megabases.

In an effort to better characterize the induced inversions we utilized Volocity software to quantify the size of the inversions. Inversions were observed as small as 0.6 Mb and as large as 90 Mb in cells exposed to 2 Gy of radiation. As seen in Figure 4 all of the exposed samples have a statistically significant shift in inversion size in both Fe ion or X-ray exposed cells, most prominently to sizes smaller than 30 Mb, p-values for Fe ions was <0.0001 and for X-ray’s was <0.01. The average size of the “false” inversion in unirradiated cells was 25 Mb. To better understand the role of LET on the size of induced inversions, inversions smaller than 30 Mb were analyzed. 30 Mb was selected based on the statistical calculations that nearly all observed “False” inversions were most likely larger then 30 Mb. As seen in Figure 4 the differences between Fe ion exposed and X-ray exposed cells becomes more evident. Induced inversions in both G1 and M phase Fe ion exposed cells were shown to be statistically smaller than the inversions formed in X-ray exposed cells (P value of 0.0036 and 0.0027 for G1 and M phase respectively). The average size of inversions smaller than 30 Mb for X-ray G1 exposed cells was 13.34 Mb and for M phase cells it was 12.02. When compared to Fe exposed cells the observed inversions averaged 9.9 and 8.7 Mb for G1 and M phase cells respectively. Upon analyzing the inversion sizes of both Fe ion exposed G1 and M phase cells we found the largest variation between the cell cycle phases was seen in fragments smaller than 15 Mb. We observed that M phase exposed cells produced statistically smaller inversions than G1 exposed cells.

## Discussion

It can be seen in our study that both the LET of the radiation and chromatin structure play a role not only in the induction of chromosomal inversion, but also in the size of the induced inversions. Our modified staining protocol has allowed inversions to be observed on a level not seen in previous studies. To account for inversions observed at 0 Gy we used Poisson Distribution to show that these inversions were actually two SCE events occurring on a single chromatid within a close distance to one another. The small differences between our calculated false inversions and the observed inversions at 0 Gy can be attributed to naturally occurring true inversions, which is an extremely rare event. Our data strongly correlates with prior observations, with the exception of one prior study. In this paper, it was noted that radiation induced interstitial exchanges were formed primarily by true SCE events [16]. Based on our observation of micro inversions, aberrations that were undetectable in this earlier study, and results from several other studies we believe that the majority of radiation-induced inversions are in fact true inversions and not caused by 2 SCE events. These findings highlight the importance of these micro inversions and additionally support the idea that ionizing radiation can produce inversions.

In conclusion, this study has effectively shown that the size and number of induced inversions are affected by both the LET of the radiation and the chromatin structure of the DNA. It appears that high LET radiation, Fe ions, create inversions whose size and number are directly dependent on chromatin structure, this observation was not seen in cells exposed to low LET X-rays. Additionally, we were able to show that high LET radiation was more effective at inducing inversions than the low LET radiation. Finally, the staining protocol utilized in this study was able to observe inversions smaller than previously reported, and by having this ability to observe these micro inversions allowed us to accurately record the number of induced inversions and avoid the background level of false inversions [3-5,15].

## Conclusion

We have been able to observe chromosomal inversions in a finer detail then prior papers have been able to achieve. We modified a traditional Giemsa staining approach by utilizing a fluorescent probe to identify inversions as small as 0.6 Mb. Using this approach we were able to see changes in not only the overall number of radiation induced inversion, but also a change in the size of the induced inversions. In this study we have shown that the cell cycle only effects the number and size of induced inversions if the cells were exposed to high LET radiation. It was seen that both G1 and M phase cells exposed to Fe ions had more and smaller inversions than X-ray exposed cells. Additionally, there was a difference between G1 and M phase Fe ion exposed cells, unlike X-ray exposed cells. Exposure to Fe ions produced more inversions in G1 cells, however the overall size is larger than M phase exposed cells.

## Material and methods

### Cell lines

Chinese Hamster Ovary 10B2 (CHO10B2) cells were kindly supplied from Dr. Joel Bedford at Colorado State University (Fort Collins, CO). Cells were cultured in MEM-alpha (Gibco, Indianapolis, IN) supplemented with 10% fetal bovine serum (FBS, Sigma, St Louis, MO) and 1% antibiotics and antimycotics (Gibco), and they were maintained at 37°C in a humidified atmosphere of 5% CO_2_ in air. The CHO10B2 cells where cultured for 1 cycle, 12 hours, with 1 μM BrdU (Sigma) to ensure uniform incorporation into the newly synthesized DNA and then harvested either in the G1 or M phase of the cell cycle by mitotic shake off [17-19]. CHO10B2 cells were chosen due to they short division time and the ability to effectively synchronize the cell population into either G1 or M phase.

### Synchronization

Cells were synchronized into either G1 or M phase via a classic mitotic shake-off procedure and only cells with a mitotic index of 90% or higher were used [20-22]. For collection of G1 synchronized cells, the collected mitotic cells were incubated for 2 hours at 37°C to allow for the cells to proceed from M phase to G1. For collection of M phase synchronized cells the mitotic cells were collected immediately prior to irradiation and transferred into pre-warmed T25 flasks and irradiated.

### Irradiation sources

Cells were irradiated with X-rays using a TITAN X-ray generator (Shimadzu, Tokyo, Japan) using 5 mm Al and Cu filters at 200 kVp and 20 mA. The dose rate was approximately 1 Gy/min for X-ray. Cells were also irradiated using accelerated iron-ions at HIMAC (Heavy Ion Medical Accelerator in Chiba), the National Institute of Radiological Sciences in Chiba, Japan, which have 500 MeV/nucleon of initial energy and 200 keV/μm of LET.

### Metaphase chromosome preparation

Cells were sub-cultured immediately after irradiation and 0.1 μg/ml of colcemid was added to the flask of cells for 18 hours. The cells were harvested during the first post-irradiated metaphase. Cells were trypsinized and then suspended in 6 ml of a 75 mM KCl solution warmed to 37°C and placed in a 37°C water bath for 20 minutes. Carnoy’s solution (3:1 methanol to acetic acid) was added to the samples according to the standard protocol. The fixed cells were dropped onto slides. These were set aside and allowed to dry until the Carnoy’s solution had evaporated, roughly 4–5 minutes [23].

### Staining

Chromosomes where denatured for 3 minutes in an 80°Celsius 70% formamide in 2× saline-sodium citrate (SSC) solution than washed in 2× SSC for 10 minutes [15]. The chromosomes where stained with 1/1000 anti-BrdU antibody (BD Biosciences, San Jose, CA) for 2 hours and than a secondary Alexa Fluor 488 (Invitrogen, Washington, D.C.) antibody was applied for 2 hours. The chromosomes where counter stained with Prolong Gold Antifade with 4,6-diamidino-2-phenylindole (DAPI) (Invitrogen).

### Image analysis

Olympus BX51 fluorescence microscope (Olympus, Tokyo, Japan) equipped with Q-imaging Aqua cooled CCD camera (Q-imaging, Surrey, BC, Canada) was used for image capture. DAPI and anti-BrdU signals where merged using ImageJ software (National Institute of Health, Maryland, USA).

### Measurements of inversions

The size of the inversions was determined using the image analysis software Volocity (PerkinElmer, Waltham, MA). Using Volocity we measured the pixel intensity of each inversion and the total pixel intensity of all 21 chromosomes in each cell. To determine the size of the inversion we compared the total pixel intensity to the CHO genome size, roughly 2.45 gigabases. This allowed us to determine the number of basepairs per pixel for each metaphase spread.

### Statistical analysis

Statistical comparison of mean values was performed using a two tailed t-test. Differences with a P-value of <0.05 were considered to indicate a statistically significant result. Error bars indicate the standard error of the means. Confidence interval values were calculated by Prism 5™ software (GraphPad, La Jolla, CA, USA). Induction rates were considered statistically similar if the slope fell within the 95% confidence interval of compared slope.

### Classification of aberrations

Inversions where categorized into two groups, inversions and micro inversions. All interstitial exchanges were classified as inversions; these include both true and false inversions. The total inversions where further categorized by size. Any inversion that was smaller than the width of a chromatid was considered a micro inversion, all other inversions remained categorized as an inversion. As discussed later in the paper, induced micro inversions can be considered with confidence to be true inversions.

## Competing interests

The authors declare that they have no competing interests.

## Authors’ contributions

IC carried out the immunofluorescence imaging, analyzed both Giemsa and immunofluorescence images, conducted the statistical analyses, and drafted the manuscript. MG carried out the Giemsa imaging and aided in the editing of the manuscript. TK an FA conceived of the study, participated in its design and coordination, and helped to draft the manuscript. All authors read and approved the final manuscript.
